# Purine Metabolism–Related Pathogenic Genes in Trigeminal Neuralgia: A Multiomics Mendelian Randomization Study

**DOI:** 10.1155/prm/1640039

**Published:** 2026-08-06

**Authors:** Simin Shen, Jiyan Li, Zheng Liu, Xili Zhang, Xiangming Li, Peiyao Li, Yaping Liu, Libiao Yuan, Ying He, Zhangxiang Huang

**Affiliations:** ^1^ Department of Pain, First Affiliated Hospital of Kunming Medical University, Kunming 650000, Yunnan, China, kmmc.cn; ^2^ Yunnan Technology and Business University, Kunming 651701, Yunnan, China; ^3^ Eye Department, Kunming Tongren Hospital, Kunming 650000, Yunnan, China; ^4^ Department of Pain Treatment, Lincang People’s Hospital, Lincang 677000, Yunnan, China

**Keywords:** genetic variants, GWAS, Mendelian randomization, multiomics, purine, trigeminal neuralgia

## Abstract

**Purpose:**

To investigate the association between purine metabolism–related genes and trigeminal neuralgia (TN) using multiomics approaches.

**Methods:**

A multiomics summary‐data‐based Mendelian randomization (SMR) approach was applied to investigate associations between purine metabolism–related genes and TN. Genome‐wide association study (GWAS) data from FinnGen R10 were integrated with blood‐derived DNA methylation quantitative trait loci (mQTL), expression quantitative trait loci (eQTL), and protein quantitative trait loci (pQTL) datasets. Associations were evaluated by colocalization and replication in UKBB (G6_TRINEU) and FinnGen R12. Drug‐target MR analysis used blood eQTL variants as proxies for gene expression. GEO transcriptomic and single‐cell RNA sequencing (scRNA‐seq) datasets and GTEx brain tissue eQTL data provided expression and tissue‐level validation. Functional enrichment, protein–protein interaction network, and druggability analyses were performed.

**Results:**

SMR analysis identified 238 mQTLs (127 genes), 30 eQTLs, and 3 pQTLs associated with TN. Colocalization supported 96 mQTLs (23 genes), 18 eQTLs, and 3 pQTLs, with 42 mQTLs and the *CNOT7* eQTL validated in UKBB. *POMGNT2* showed genetically predicted associations with TN at both gene and protein levels. Integrative analysis highlighted *ATP6V0E2, PLAT,* and *VKORC1* with evidence of epigenetic regulation. Drug‐target MR provided supportive evidence that genetically proxied expression of *CNOT7* and *PLAT* may be associated with lower TN risk. Transcriptomic validation showed downregulation of *ATP6V0E2, PLAT,* and *CNOT7* after trigeminal nerve injury. Brain eQTL analysis supported the association of *CNOT7* with TN across multiple brain regions. scRNA‐seq revealed cell‐type‐specific expression patterns of prioritized genes in trigeminal ganglion tissues. Functional enrichment indicated involvement in stress response, metabolism, and genome maintenance pathways. PPI analysis identified six hub genes: *ABCB1, COMT, GFM1, MDH2, NRAS,* and *TXN*. Druggability analysis showed that *ATP6V0E2, PLAT,* and *VKORC1* are targetable by existing drugs.

**Conclusion:**

This study prioritizes purine metabolism–linked genes showing genetically supported associations with TN and highlights potential biological pathways and therapeutic targets.

## 1. Introduction

Trigeminal neuralgia (TN) is a prevalent cranial neuropathic disorder characterized by unilateral, brief, and severe facial pain episodes, often described as “electrical shock”‐like in nature [1]. The incidence of TN ranges from 0.16% to 0.3%, with a higher prevalence among females over the age of 40 [2]. Current treatments include pharmacotherapy and surgical intervention, both of which are limited in efficacy. In particular, drug therapy lacks robust clinical evidence, has significant side effects, may not be effective in the long term, and can lead to drug resistance in some patients. Surgical procedures carry risks of complications and potential for recurrence [3]. Although the precise pathogenesis of TN remains unclear, research suggests that its progression involves processes such as neuronal damage and nerve regeneration [4]. In these processes, purine metabolism disorder plays a key role [5]. When the trigeminal nerve is damaged, purines may lead to an increase in neuronal hyperexcitability through the activation of these receptors, thereby enhancing pain perception—a phenomenon known as central sensitization, which is a significant pathological mechanism in TN [6].

Disruptions in purine metabolism can alter blood uric acid levels. Uric acid, the end product of purine metabolism, serves as an antioxidant at normal concentrations, helping to remove free radicals and protect the body from oxidative stress damage [7]. Clinical studies have indicated that patients with TN exhibit significantly reduced levels of uric acid [8]. Low serum uric acid levels have been associated with an increased risk of TN and more severe clinical symptoms, suggesting that appropriately increasing uric acid levels might help prevent TN [9]. Despite recognizing the key role of purine metabolism disorder in the pathogenesis of TN, the specific genes involved in this process remain to be elucidated.

Mendelian randomization (MR) uses genetic variants as instrumental variables to evaluate potential associations between exposures and outcomes while reducing confounding from environmental factors [10]. Recent advances in multiomics integration and computational approaches have enabled the identification of disease‐associated genes across multiple molecular layers, providing a systematic framework for investigating complex disease mechanisms [11, 12]. This study employed a summary‐data‐based Mendelian randomization (SMR approach), integrating Genome‐wide association study (GWAS), methylation QTL (mQTL), expression QTL (eQTL), and protein QTL (pQTL) data, to explore the associations between purine metabolism–related genes and TN. SMR prioritizes genes showing regulatory variant associations with disease risk [13]. The HEIDI test distinguishes pleiotropic associations from linkage effects. This multiomics strategy enables the investigation of TN‐related genetic signals across multiple molecular layers, including methylation, gene expression, and protein levels.

In this study, we adopted a broad definition of “purine metabolism–linked genes.” The initial gene set obtained from GeneCards includes not only enzymes directly involved in purine synthesis, degradation, and transport but also genes functionally connected to purine biology through ATP‐dependent processes, oxidative stress, neuroinflammation, and neuronal excitability. Therefore, the genes prioritized in our analysis may reflect both direct metabolic components and indirect functional regulators of purine‐related pathways. This conceptual framework distinguishes direct biochemical roles from indirect functional associations in the interpretation of prioritized genes.

The aim of this study was to investigate the associations between purine metabolism–related genes and TN using the SMR method. This approach evaluates the potential involvement of these genes in TN at multiple molecular levels and explores the role of purine metabolism dysregulation in TN pathogenesis. The findings may provide new insights into the mechanisms underlying TN and support the development of targeted therapeutic strategies.

## 2. Materials and Methods

### 2.1. Study Design

**FIGURE 1:**
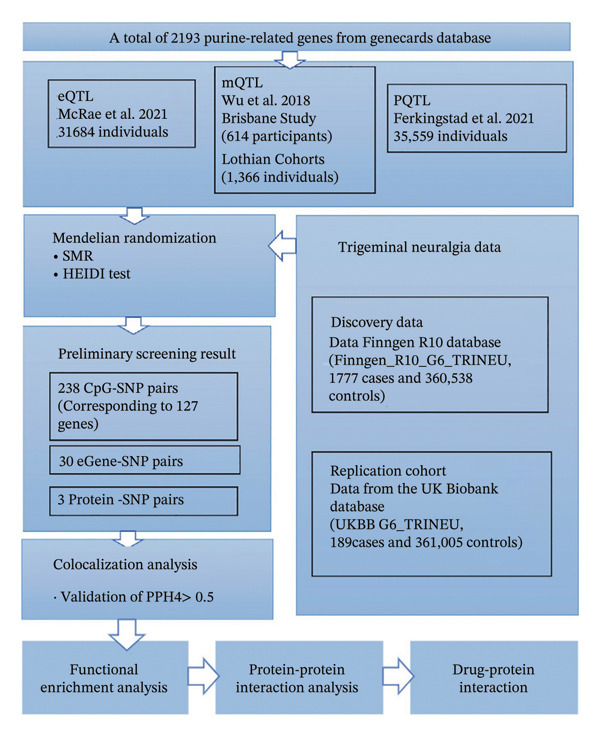
Study design flowchart.

This study was designed according to the STROBE‐MR reporting guidelines [14] and used an MR approach to explore the associations between purine metabolism–related genes and TN. The approach included genetic association analysis using SMR and HEIDI tests, followed by colocalization analysis to evaluate whether GWAS and QTL signals shared the same underlying variant. The study design, workflow, and analytical methods are detailed in Figure 1. Because the study was based on publicly available data, ethical approval was not required.

### 2.2. Data Sources

A total of 2193 purine metabolism–related genes were acquired from the GeneCards database. The discovery dataset for TN was obtained from the Finngen R10 GWAS (ID: Finngen_R10_G6_TRINEU), including 1777 cases and 360,538 controls of Finnish ancestry. For replication, GWAS data from the UK Biobank (ID: UKBB G6_TRINEU) were used, comprising 189 cases and 361,005 controls of predominantly European ancestry. An updated FinnGen R12 TN GWAS dataset (2226 cases and 435,371 controls) was further used for gene‐level replication.

Blood eQTL summary data from 31,684 individuals of predominantly European ancestry were obtained from the eQTLGen database [15] and were used for SMR analysis and drug‐target MR analyses as genetic proxies for gene expression. Blood mQTL summary data were obtained from a meta‐analysis of two European cohorts: the Brisbane Systems Genetics Study (*n* = 614) and the Lothian Birth Cohorts (*n* = 1366) [13]. Blood pQTL summary data were obtained from a large‐scale plasma proteomic study, including 35,559 Icelandic individuals [16]. To provide tissue‐relevant validation, brain tissue eQTL data from the Genotype‐Tissue Expression Project (GTEx; https://gtexportal.org) were used to perform SMR analyses for the prioritized genes. The GTEx dataset includes gene expression and genotype data across multiple brain regions, with sample sizes ranging from 114 to 209 donors per tissue [17].

To further assess expression changes of prioritized genes, transcriptomic datasets GSE162284 [18] and GSE256472, derived from mouse TN models, were obtained from the Gene Expression Omnibus (GEO) database. In GSE162284, differential expression was analyzed across three time points: baseline (D0), 3 days (D3), and 21 days (D21) after injury, including the comparisons D3 vs D0, D21 vs D0, and D21 vs D3. In addition, the GSE197289 single‐cell RNA sequencing (scRNA‐seq) dataset from mouse trigeminal ganglion tissue was used to examine the cellular distribution of the prioritized genes. Cell‐type annotations were adopted from the original study [19], including major neuronal subtypes and non‐neuronal populations such as satellite glia, Schwann cells, fibroblasts, immune cells, and vascular cells.

### 2.3. SMR Analysis

We employed the SMR software tool version 1.3.1 to investigate the associations between methylation, expression, and protein abundance of purine metabolism–related genes and TN using SMR and HEIDI tests [13]. Prior to SMR analysis, mQTL, eQTL, and pQTL datasets were filtered to retain variants with minor allele frequency (MAF) > 0.01 and *cis*‐acting effects located within ±1 Mb of the corresponding probe. SMR analysis was then performed using the top associated *cis*‐QTLs with genome‐wide significance (p < 5.0 × 10^−8^) as instrumental variables [20]. SNPs with allele frequency differences greater than 0.2 between the LD reference panel, QTL summary data, and outcome summary data were excluded, with a maximum of 0.05 allowed for SNPs with allele frequency differences.

A multi‐SNP SMR approach (‐SMR‐multi) was applied to account for multiple variants at each QTL locus. To minimize confounding due to linkage or horizontal pleiotropy, the HEIDI test was performed, and associations with P‐HEIDI ≤ 0.01 were excluded. Associations meeting nominal significance thresholds (P‐SMR_multi < 0.05 and P‐SMR < 0.05) and P‐HEIDI > 0.01 underwent colocalization analysis to evaluate whether QTL signals (eQTLs and mQTLs) and TN GWAS loci shared a common underlying variant, thereby reducing false‐positive findings.

Beyond exploring the associations between QTLs and TN, we further investigated the relationship between mQTLs and eQTLs by treating mQTLs as the exposure and eQTLs as the outcome. The key results obtained for mQTLs and eQTLs with respect to TN were considered important signals of the regulatory relationships between mQTLs and eQTLs [21].

### 2.4. Colocalization

Colocalization analysis, using the R package “coloc,” identified shared variants between purine‐related *cis*‐QTLs and TN [22]. When GWAS signals and QTLs were found to colocalize, we hypothesized that the locus on the GWAS signal may influence the phenotype by altering gene‐related biological processes (BP). In the colocalization analyses, five different posterior probabilities were reported, which corresponded to the five exclusionary hypotheses [23]: H0 (no trait genetically associated with an SNP), H1 (only trait 1 genetically associated with an SNP), H2 (only trait 2 genetically associated with an SNP), H3 (two traits are associated with an SNP, but using different causal variables), and H4 (two traits are associated with an SNP and share a dependent variable). Colocalization analysis was performed on SNPs within 1000 kb of the top *cis*‐QTLs colocalization region, with coloc parameter P12 set to 5 × 10^−5^ and PPH4 > 0.5 as the posterior probability threshold for colocalization [24].

All statistical analyses were performed using R (v4.3.0). The R package “ggplot2” was used for Manhattan plot generation, and “forestplot” was used for forest plot generation. The code for SMR Locus Plot and SMR Effect Plot was adapted from Zhu et al. [25].

### 2.5. Drug‐Target MR Analysis

To further evaluate SMR‐prioritized genes, drug‐target MR was conducted using genetically predicted gene expression as the exposure and TN risk as the outcome. Blood eQTL variants from the eQTLGen consortium (*n* = 31,684) were used as genetic proxies for gene expression. TN GWAS summary statistics from the FinnGen R12 dataset (2226 cases and 435,371 controls) were used as the outcome data. SNPs located within ±300 kb of each target gene were selected as instrumental variables if they were significantly associated with gene expression (false discovery rate [FDR] < 0.05, controlling for multiple testing across probes) and had a MAF > 0.01. Independent variants were obtained using LD clumping (*r* ^2^ < 0.3, 500‐kb window), and weak instruments were excluded using an *F*‐statistic threshold > 10. Causal effects were primarily estimated using the inverse variance weighted (IVW) method, with MR‐Egger, weighted median, and weighted mode applied as complementary approaches. Heterogeneity and horizontal pleiotropy were evaluated using Cochran’s *Q* test, the MR‐Egger intercept, and MR‐PRESSO. Directionality was assessed using the Steiger test. Sensitivity analyses included leave‐one‐out testing and radial MR to detect potential outliers. All analyses were conducted in R (v4.3.0) using the TwoSampleMR and RadialMR packages.

### 2.6. Functional Enrichment

Gene Ontology (GO) enrichment analysis was conducted to explore the BP, molecular functions (MF), and cellular components (CC) of the key genes associated with TN risk. Kyoto Encyclopedia of Genes and Genomes (KEGG) was utilized to investigate the pathway in which these genes were involved. The package “Clusterprofiler” was used for GO and KEGG analysis and visualization. Terms with Benjamini–Hochberg FDR < 0.05 were considered statistically significant.

### 2.7. Protein–Protein Interaction (PPI) Network

The STRING database (https://cn.string-db.org/) was employed to construct a PPI network based on TN‐associated genes identified from multiomics analyses. The topological features of the nodes within this PPI network were calculated with seven algorithms, including degree, closeness, and radiality [26]. The top 10 proteins were identified based on each algorithm, and the hub proteins were defined as those that overlapped across all seven algorithms.

### 2.8. Druggability Analysis

To assess the therapeutic potential of key targets, candidate drugs were predicted using the DSigDB database [27].

### 2.9. Statistical Thresholds and Multiple‐Testing Correction

SMR analyses applied P‐SMR < 0.05 and P‐HEIDI > 0.01 as nominal significance criteria. Colocalization analyses used PPH4 > 0.5 as evidence supporting shared causal variants. Differential expression analyses used Benjamini–Hochberg adjusted *p* < 0.05. GO and KEGG enrichment analyses used FDR < 0.05. Drug‐target MR analyses selected eQTL instruments with FDR < 0.05 and applied IVW as the primary method, with complementary MR models for sensitivity assessment. These thresholds were predefined and consistently applied across all analyses.

## 3. Results

### 3.1. Integration of TN GWAS and Purine Metabolism–Related Blood mQTL Data

**FIGURE 2:**
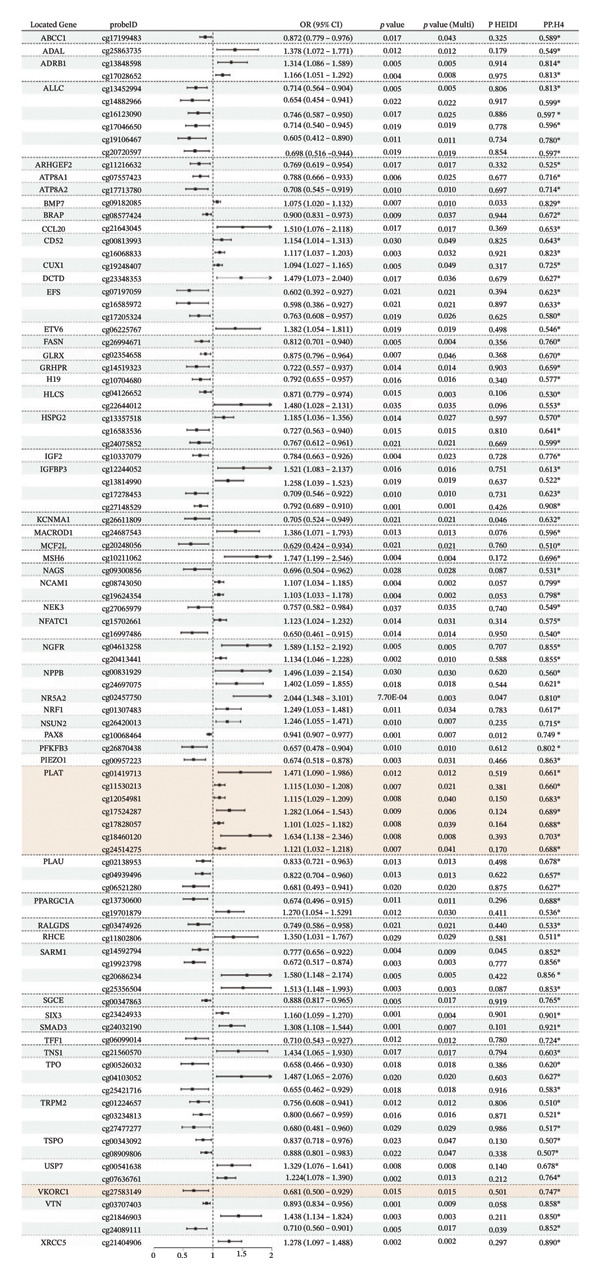
Forest plot of SMR association results for mQTLs validated by colocalization analysis. Prioritized genes (*ATP6V0E2, PLAT, VKORC1, CNOT7,* and *POMGNT2*) are shown in bold to improve readability; full SMR results are provided in the supporting figures and tables.

The SMR analysis identified 238 methylation sites (corresponding to 127 genes) associated with TN (Table 1, Table S1, P‐SMR_multi < 0.05 & P‐SMR < 0.05 & P‐HEIDI > 0.01). Within the colocalization region windows of SNPs corresponding to 23 genes, there were 96 methylation sites associated with TN (Figure 2, PPH4 > 0.5). A total of 15 genes corresponding to 42 methylation sites were validated in the UKBB G6_TRINEU cohort (Table 2, Table S2).

**TABLE 1:** SMR analysis results of mQTLs in the Finngen_R10_G6_TRINEU Cohort (PP.H4 > 0.5).

ProbeID	Symbol	P‐SMR	P‐SMR_multi	OR (95% CI)	PP.H4
cg25863735	ADAL	0.01245	0.012453	1.378 (1.072–1.771)	0.548717
cg14519323	GRHPR	0.01422	0.014221	0.722 (0.557–0.937)	0.65894
cg24687543	MACROD1	0.01304	0.013035	1.386 (1.071–1.793)	0.596073
cg01307483	NRF1	0.01075	0.03448	1.249 (1.053–1.481)	0.617497
cg10704680	H19	0.01559	0.015585	0.792 (0.655–0.957)	0.577251
cg19624354	NCAM1	0.00355	0.002477	1.103 (1.033–1.178)	0.798243
cg08743050	NCAM1	0.00357	0.002073	1.107 (1.034–1.185)	0.799016
cg06225767	ETV6	0.01907	0.01907	1.382 (1.054–1.811)	0.546325
cg20413441	NGFR	0.00217	0.010137	1.134 (1.046–1.228)	0.855118
cg04613258	NGFR	0.00478	0.004784	1.589 (1.152–2.192)	0.855111
cg07636761	USP7	0.00184	0.013294	1.224 (1.078–1.39)	0.763626
cg00541638	USP7	0.00832	0.008315	1.329 (1.076–1.641)	0.678261
cg26611809	KCNMA1	0.0213	0.021304	0.705 (0.524–0.949)	0.632449
cg02138953	PLAU	0.01324	0.013241	0.833 (0.721–0.963)	0.67808
cg04939496	PLAU	0.01348	0.013481	0.822 (0.704–0.96)	0.65746
cg06521280	PLAU	0.01969	0.01969	0.681 (0.493–0.941)	0.627474
cg00347863	SGCE	0.00489	0.017395	0.888 (0.817–0.965)	0.765013
cg13357518	HSPG2	0.01352	0.026534	1.185 (1.036–1.356)	0.570456
cg24075852	HSPG2	0.02091	0.020911	0.767 (0.612–0.961)	0.599438
cg16583536	HSPG2	0.01498	0.014983	0.727 (0.563–0.94)	0.641408
cg09300856	NAGS	0.02819	0.028185	0.696 (0.504–0.962)	0.530916
cg15702661	NFATC1	0.01381	0.030808	1.123 (1.024–1.232)	0.574641
cg16997486	NFATC1	0.01372	0.013809	0.65 (0.461–0.915)	0.540176
cg21404906	XRCC5	0.00164	0.001642	1.278 (1.097–1.488)	0.890333
cg21846903	VTN	0.00277	0.002767	1.438 (1.134–1.824)	0.850166
cg03707403	VTN	0.00122	0.008558	0.893 (0.834–0.956)	0.857605
cg24089111	VTN	0.00482	0.016875	0.71 (0.56–0.901)	0.851905
cg19923798	SARM1	0.00302	0.003018	0.672 (0.517–0.874)	0.85596
cg20686234	SARM1	0.00503	0.005027	1.58 (1.148–2.174)	0.855728
cg14592794	SARM1	0.00376	0.008729	0.777 (0.656–0.922)	0.852369
cg25356504	SARM1	0.00324	0.003236	1.513 (1.148–1.993)	0.852678
cg00813993	CD52	0.02986	0.049396	1.154 (1.014–1.313)	0.643332
cg16068833	CD52	0.0034	0.031626	1.117 (1.037–1.203)	0.822623
cg10337079	IGF2	0.00428	0.023339	0.784 (0.663–0.926)	0.77616
cg04126652	HLCS	0.01509	0.003166	0.871 (0.779–0.974)	0.579831
cg22644012	HLCS	0.03481	0.034808	1.48 (1.028–2.131)	0.55301
cg26994671	FASN	0.00547	0.004269	0.812 (0.701–0.94)	0.759713
cg01224657	TRPM2	0.01214	0.012144	0.756 (0.608–0.941)	0.510035
cg03234813	TRPM2	0.01602	0.016017	0.8 (0.667–0.959)	0.521263
cg27477277	TRPM2	0.02858	0.028581	0.68 (0.481–0.96)	0.517441
cg10211062	MSH6	0.00366	0.003659	1.747 (1.199–2.546)	0.695647
cg23424933	SIX3	0.00142	0.003824	1.16 (1.059–1.27)	0.901166
cg13452994	ALLC	0.00517	0.005171	0.714 (0.564–0.904)	0.813059
cg16123090	ALLC	0.01733	0.024824	0.746 (0.587–0.95)	0.596632
cg20720597	ALLC	0.01944	0.019445	0.698 (0.516–0.944)	0.597451
cg14882966	ALLC	0.02213	0.022128	0.654 (0.454–0.941)	0.59949
cg17046650	ALLC	0.01864	0.018637	0.714 (0.54–0.945)	0.596384
cg19106467	ALLC	0.01064	0.010641	0.605 (0.412–0.89)	0.780402
cg00957223	PIEZO1	0.00341	0.003075	0.674 (0.518–0.878)	0.862625
cg24697075	NPPB	0.01823	0.018234	1.402 (1.059–1.855)	0.621057
cg00831929	NPPB	0.03043	0.030431	1.496 (1.039–2.154)	0.559738
cg19701879	PPARGC1A	0.01186	0.029857	1.27 (1.054–1.529)	0.536036
cg13730600	PPARGC1A	0.01141	0.011409	0.674 (0.496–0.915)	0.687676
cg00343092	TSPO	0.0232	0.046964	0.837 (0.718–0.976)	0.506803
cg08909806	TSPO	0.02214	0.042488	0.888 (0.801–0.983)	0.507124
cg12244052	IGFBP3	0.01557	0.015568	1.521 (1.083–2.137)	0.612974
cg17278453	IGFBP3	0.01022	0.010216	0.709 (0.546–0.922)	0.623273
cg13814990	IGFBP3	0.01853	0.018535	1.258 (1.039–1.523)	0.522448
cg27148529	IGFBP3	0.00103	0.001253	0.792 (0.689–0.91)	0.908336
cg04103052	TPO	0.01983	0.019825	1.487 (1.065–2.076)	0.626883
cg25421716	TPO	0.01772	0.017721	0.655 (0.462–0.929)	0.583151
cg00526032	TPO	0.01765	0.017648	0.658 (0.466–0.93)	0.620053
cg09182085	BMP7	0.00683	0.009983	1.075 (1.02–1.132)	0.828708
cg10068464	PAX8	0.00143	0.006518	0.941 (0.907–0.977)	0.748812
cg06099014	TFF1	0.01174	0.011742	0.71 (0.543–0.927)	0.72384
cg16585972	EFS	0.02142	0.021419	0.598 (0.386–0.927)	0.632646
cg17205324	EFS	0.01929	0.025842	0.763 (0.608–0.957)	0.579659
cg07197059	EFS	0.02106	0.021058	0.602 (0.392–0.927)	0.623399
cg02354658	GLRX	0.00652	0.04626	0.876 (0.796–0.964)	0.669755
cg23348353	DCTD	0.01699	0.036087	1.479 (1.073–2.04)	0.627126
cg27583149	VKORC1	0.0153	0.015302	0.681 (0.5–0.929)	0.747497
cg11216632	ARHGEF2	0.01717	0.017167	0.769 (0.619–0.954)	0.524755
cg17199483	ABCC1	0.01704	0.042957	0.872 (0.779–0.976)	0.589101
cg07557423	ATP8A1	0.00569	0.02475	0.788 (0.666–0.933)	0.716314
cg13848598	ADRB1	0.00495	0.004949	1.314 (1.086–1.589)	0.813646
cg17028652	ADRB1	0.00358	0.008429	1.166 (1.051–1.292)	0.813006
cg20248056	MCF2L	0.02141	0.021405	0.629 (0.424–0.934)	0.509666
cg03474926	RALGDS	0.02138	0.021379	0.749 (0.586–0.958)	0.532693
cg26420013	NSUN2	0.00957	0.007067	1.246 (1.055–1.471)	0.715477
cg21643045	CCL20	0.01721	0.017214	1.51 (1.076–2.118)	0.653408
cg17713780	ATP8A2	0.00959	0.009591	0.708 (0.545–0.919)	0.713707
cg24032190	SMAD3	0.00149	0.007273	1.308 (1.108–1.544)	0.920556
cg17524287	PLAT	0.00887	0.005649	1.282 (1.064–1.543)	0.689014
cg24514275	PLAT	0.00705	0.04112	1.121 (1.032–1.218)	0.687535
cg12054981	PLAT	0.00801	0.03964	1.115 (1.029–1.209)	0.682788
cg17828057	PLAT	0.00793	0.03882	1.101 (1.025–1.182)	0.687627
cg11530213	PLAT	0.00745	0.020892	1.115 (1.03–1.208)	0.659725
cg01419713	PLAT	0.01165	0.011652	1.471 (1.09–1.986)	0.660704
cg18460120	PLAT	0.00786	0.007857	1.634 (1.138–2.346)	0.7027
cg19248407	CUX1	0.0051	0.048504	1.094 (1.027–1.165)	0.72547
cg02457750	NR5A2	0.00077	0.003123	2.044 (1.348–3.101)	0.810359
cg26870438	PFKFB3	0.00992	0.009924	0.657 (0.478–0.904)	0.801548
cg27065979	NEK3	0.03743	0.034915	0.757 (0.582–0.984)	0.548606
cg11802806	RHCE	0.02906	0.029055	1.35 (1.031–1.767)	0.511047
cg21560570	TNS1	0.01738	0.017379	1.434 (1.065–1.93)	0.603206
cg08577424	BRAP	0.00863	0.037007	0.9 (0.831–0.973)	0.671958

**TABLE 2:** mQTLs‐GWAS validation results: mQTLs SMR Analysis by UKBB_ G6_TRINEU cohort.

ProbeID	Symbol	P‐SMR	P‐SMR_multi	OR (95% CI)
cg14536784	PDE4A	0.00914	0.009136419	1.001 (1, 1.001)
cg11863536	PDE4A	0.00704	0.02486525	1.001 (1, 1.001)
cg01857838	PDE10A	0.04488	0.04487762	1 (1, 1.001)
cg24687543	MACROD1	0.02088	0.02087711	1 (0.999, 1)
cg26467918	MACROD1	0.03548	0.00927181	1 (0.999, 1)
cg01307483	NRF1	0.01591	0.04952292	1 (1, 1.001)
cg19273378	KCNMA1	0.01424	0.01424122	0.999 (0.999, 1)
cg19850275	PLAU	0.00463	0.004626286	0.999 (0.998, 1)
cg06829584	PLAU	0.02162	0.0216166	1 (0.999, 1)
cg02138953	PLAU	0.01171	0.01171377	1 (0.999, 1)
cg04939496	PLAU	0.01194	0.01194007	1 (0.999, 1)
cg06521280	PLAU	0.02619	0.0261934	0.999 (0.999, 1)
cg09300856	NAGS	0.02237	0.02236575	1.001 (1, 1.001)
cg04196345	NAGS	0.01914	0.013139	1 (0.999, 1)
cg17406359	TRPM2	0.01053	0.0105256	1.001 (1, 1.001)
cg08191854	TRPM2	0.01415	0.01415031	0.999 (0.999, 1)
cg07806328	TRPM2	0.03207	0.03206734	1 (0.999, 1)
cg01224657	TRPM2	0.03081	0.0308082	1 (1, 1.001)
cg27328431	TRPM2	0.02951	0.0266477	1 (1, 1.001)
cg03234813	TRPM2	0.03072	0.03072109	1 (1, 1.001)
cg13452994	ALLC	0.0145	0.01449801	1 (1, 1.001)
cg16123090	ALLC	0.02721	0.03664738	1 (1, 1.001)
cg20720597	ALLC	0.02968	0.02968232	1.001 (1, 1.001)
cg14882966	ALLC	0.03277	0.03277042	1.001 (1, 1.001)
cg17046650	ALLC	0.02874	0.02874033	1 (1, 1.001)
cg15681466	ALLC	0.00911	0.009105781	0.999 (0.999, 1)
cg01332683	ABCC1	0.018	0.01799798	0.999 (0.999, 1)
cg23137531	MCF2L	0.04603	0.04603215	0.999 (0.999, 1)
cg14864103	MCF2L	0.04233	0.04233415	1 (0.999, 1)
cg05437446	MCF2L	0.0442	0.04420448	0.999 (0.999, 1)
cg25745937	MCF2L	0.03032	0.03031605	1 (0.999, 1)
cg01248385	MCF2L	0.04214	0.04214095	1.001 (1, 1.001)
cg14765414	MCF2L	0.02734	0.02733857	1.001 (1, 1.001)
cg12597614	MCF2L	0.03237	0.04060868	1 (1, 1)
cg04040317	MCF2L	0.03469	0.03469391	1.001 (1, 1.001)
cg22981175	MCF2L	0.01777	0.01776565	1 (1, 1.001)
cg16960046	EGFR	0.0403	0.04029682	1.001 (1, 1.001)
cg13490352	EGFR	0.03234	0.0151596	1 (1, 1.001)
cg25866552	EGFR	0.04885	0.04884604	1.001 (1, 1.001)
cg19193595	SMAD3	0.01819	0.01819016	0.999 (0.999, 1)
cg24283621	PFKFB3	0.02853	0.02852902	0.999 (0.999, 1)
cg05115468	SNHG1	0.04664	0.0466379	1 (0.999, 1)

### 3.2. Integration of TN GWAS and Purine Metabolism–Related Blood eQTL Data

**FIGURE 3:**
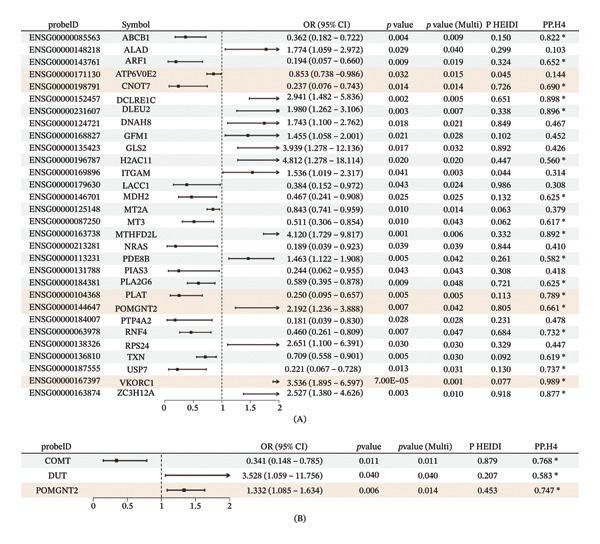
Forest plot. (A) SMR analysis results for eQTLs in the Finngen_R10_G6_TRINEU cohort. (B) SMR analysis results for pQTLs in the Finngen_R10_G6_TRINEU cohort. Prioritized genes (*ATP6V0E2, PLAT, VKORC1, CNOT7,* and *POMGNT2*) are shown in bold to improve readability; full SMR results are provided in the supporting figures and tables.

The SMR analysis identified 30 genes associated with TN (Table 3, Table S3, P‐SMR_multi < 0.05 & P‐SMR < 0.05 & P‐HEIDI > 0.01). Among these, the expression levels of 14 genes (*DCLRE1C, DNAH8, ITGAM, PDE8B, DLEU2, ALAD, MTHFD2L, VKORC1, RPS24, H2AC11, GLS2, GFM1, POMGNT2, ZC3H12A*) were positively associated with the risk of TN, while the remaining 16 genes were negatively associated. Within the colocalization region windows of SNPs corresponding to 18 genes, there were sites associated with TN (Figure 3A, PPH4 > 0.5). *CNOT7* associated with TN risk was validated in the UKBB G6_TRINEU cohort (Table 4, Table S4).

**TABLE 3:** SMR analysis results of eQTLs in the Finngen_R10_G6_TRINEU cohort.

ProbeID	Symbol	P‐SMR	P‐SMR_multi	OR (95% CI)	PP.H4
ENSG00000179630	LACC1	0.04332	0.024433	0.384 (0.152–0.972)	0.307664
ENSG00000152457	DCLRE1C	0.00203	0.005076	2.941 (1.482–5.836)	0.898048
ENSG00000085563	ABCB1	0.00394	0.008605	0.362 (0.182–0.722)	0.822439
ENSG00000124721	DNAH8	0.01794	0.020905	1.743 (1.1–2.762)	0.466901
ENSG00000169896	ITGAM	0.04051	0.002686	1.536 (1.019–2.317)	0.314182
ENSG00000113231	PDE8B	0.00499	0.041518	1.463 (1.122–1.908)	0.582075
ENSG00000087250	MT3	0.01034	0.043247	0.511 (0.306–0.854)	0.616835
ENSG00000231607	DLEU2	0.00296	0.006565	1.98 (1.262–3.106)	0.896204
ENSG00000187555	USP7	0.01308	0.030909	0.221 (0.067–0.728)	0.737172
ENSG00000146701	MDH2	0.02485	0.024849	0.467 (0.241–0.908)	0.62478
ENSG00000198791	CNOT7	0.01351	0.013506	0.237 (0.076–0.743)	0.68965
ENSG00000143761	ARF1	0.00863	0.018731	0.194 (0.057–0.66)	0.651654
ENSG00000148218	ALAD	0.02946	0.040164	1.774 (1.059–2.972)	0.102705
ENSG00000163738	MTHFD2L	0.00139	0.005511	4.12 (1.729–9.817)	0.891759
ENSG00000136810	TXN	0.0049	0.029855	0.709 (0.558–0.901)	0.619205
ENSG00000167397	VKORC1	7.00E‐05	0.001376	3.536 (1.895–6.597)	0.98917
ENSG00000213281	NRAS	0.03945	0.039446	0.189 (0.039–0.923)	0.409785
ENSG00000138326	RPS24	0.02986	0.029861	2.651 (1.1–6.391)	0.447075
ENSG00000131788	PIAS3	0.04279	0.042788	0.244 (0.062–0.955)	0.418122
ENSG00000063978	RNF4	0.00699	0.04667	0.46 (0.261–0.809)	0.732209
ENSG00000196787	H2AC11	0.02019	0.020191	4.812 (1.278–18.114)	0.56003
ENSG00000184381	PLA2G6	0.00942	0.047694	0.589 (0.395–0.878)	0.624953
ENSG00000135423	GLS2	0.01696	0.032041	3.939 (1.278–12.136)	0.426363
ENSG00000104368	PLAT	0.00491	0.004913	0.25 (0.095–0.657)	0.789251
ENSG00000168827	GFM1	0.02091	0.028408	1.455 (1.058–2.001)	0.45153
ENSG00000125148	MT2A	0.00953	0.013773	0.843 (0.741–0.959)	0.379179
ENSG00000144647	POMGNT2	0.00728	0.041531	2.192 (1.236–3.888)	0.661246
ENSG00000184007	PTP4A2	0.02779	0.027786	0.181 (0.039–0.83)	0.477893
ENSG00000163874	ZC3H12A	0.00267	0.009722	2.527 (1.38–4.626)	0.876744
ENSG00000171130	ATP6V0E2	0.0315	0.014839	0.853 (0.738–0.986)	0.144007

**TABLE 4:** eQTLs‐GWAS validation results: eQTLs SMR Analysis by UKBB_ G6_TRINEU cohort.

ProbeID	Symbol	P‐SMR	P‐SMR_multi	OR (95% CI)
ENSG00000198791	CNOT7	0.021	0.02102528	0.998 (0.996, 1)

### 3.3. Integration of TN GWAS and Purine Metabolism–Related Blood pQTL Data

The SMR analysis identified three purine metabolism–related genes (*COMT, DUT, POMGNT2*) encoding proteins associated with TN (Figure 3B, Table 5, Table S5, P‐SMR_multi < 0.05 & P‐SMR < 0.05 & P‐HEIDI > 0.01). The protein abundance encoded by *COMT* was negatively associated with the risk of TN, while the protein abundance encoded by *DUT* and *POMGNT2* was positively associated. Notably, *POMGNT2* was associated with the disease at both gene expression and protein levels. Within the colocalization region windows of SNPs corresponding to these three genes (*COMT, DUT, POMGNT2*), all exhibited evidence of colocalization with TN (Figure S1C, PPH4 > 0.5). These findings were not validated in the UKBB G6_TRINEU cohort (Table S6).

**TABLE 5:** SMR analysis results of pQTLs in the Finngen_R10_G6_TRINEU cohort.

Symbol	P‐SMR	P‐SMR_multi	OR (95% CI)	PP.H4
COMT	0.01141	0.011407	0.341 (0.148–0.785)	0.767607
DUT	0.04004	0.040043	3.528 (1.059–11.756)	0.583175
POMGNT2	0.00619	0.014297	1.332 (1.085–1.634)	0.746694

### 3.4. SMR Integrates TN, Purine Metabolism–Related Blood mQTL, eQTL, and pQTL Data

**FIGURE 4:**
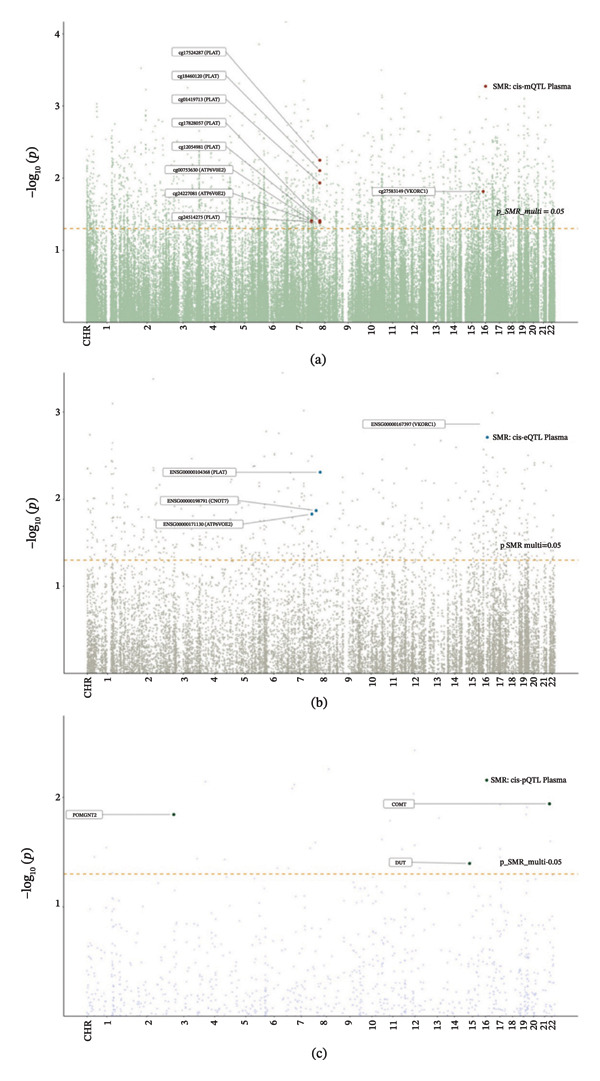
Manhattan plot of the distribution of key results on chromosomes. (A) cis‐mQTLs. (B) cis‐eQTLs; (C) cis‐pQTLs.

Based on the key findings from the TN and purine metabolism–related mQTL and eQTL data through SMR analysis, an integration of mQTL and eQTL data was conducted to further explore the regulation of blood methylation on the expression of key purine metabolism–related genes in TN (Table S7). This analysis identified nine methylation sites significantly associated with their corresponding gene expression (corresponding to three genes, *ATP6V0E2, PLAT, VKORC1*) (Table 6, P‐SMR_multi < 0.05 & P‐SMR < 0.05 & P‐HEIDI > 0.01). SMR analysis did not find any association between these three genes and protein levels. The Manhattan plot marks the distribution of these three genes and their loci on the chromosomes (Figure 4).

**TABLE 6:** mQTLs‐eQTLs SMR analysis results.

Expo_ID	Outco_Gene	Symbol	P‐SMR	P‐SMR_multi	P‐HEIDI	OR (95% CI)
cg00753630	ENSG00000171130	ATP6V0E2	5.38*E* − 13	5.38*E* − 13	0.1068918	0.149 (0.089, 0.25)
cg24227081	ENSG00000171130	ATP6V0E2	1.07*E* − 11	1.07*E* − 11	0.03121725	0.133 (0.074, 0.238)
cg17524287	ENSG00000104368	PLAT	5.55*E* − 10	7.79*E* − 10	0.1187635	0.848 (0.806, 0.894)
cg24514275	ENSG00000104368	PLAT	2.60*E* − 16	6.88*E* − 16	0.8492288	0.917 (0.899, 0.937)
cg12054981	ENSG00000104368	PLAT	3.50*E* − 16	8.42*E* − 16	0.7505533	0.921 (0.902, 0.939)
cg17828057	ENSG00000104368	PLAT	1.44*E* − 16	1.80*E* − 16	0.6358906	0.93 (0.914, 0.946)
cg01419713	ENSG00000104368	PLAT	2.23*E* − 08	2.23*E* − 08	0.01030893	0.744 (0.67, 0.825)
cg18460120	ENSG00000104368	PLAT	3.82*E* − 07	3.82*E* − 07	0.05854387	0.703 (0.614, 0.806)
cg27583149	ENSG00000167397	VKORC1	3.60*E* − 09	3.60*E* − 09	0.1870678	0.633 (0.544, 0.737)

### 3.5. Integrate Evidence From Multiple Omics Levels

Based on the above SMR analysis, *ATP6V0E2, PLAT, VKORC1, CNOT7,* and *POMGNT2* were considered high‐confidence candidate genes associated with TN. The *PLAT*‐associated CpG sites (cg17524287, cg24514275, cg12054981, cg17828057, cg01419713, and cg18460120) were positively correlated with the risk of TN. *PLAT* was negatively associated with TN risk, which was supported by colocalization (Figure S1A). cg17524287, cg24514275, cg12054981, cg17828057, cg01419713, and cg18460120 were negatively correlated with *PLAT*. Therefore, these findings suggest a potential mechanism by which higher methylation levels of cg17524287, cg24514275, cg12054981, cg17828057, cg01419713, and cg18460120 may downregulate *PLAT* gene expression, which was negatively associated with the risk of TN.

The *VKORC1* corresponding site cg27583149 in the mQTL data was negatively correlated with the risk of TN. *VKORC1* was positively associated with TN risk, which was supported by colocalization (Figure S1B). The *VKORC1* corresponding site cg27583149 was negatively correlated with *VKORC1* in the mQTL‐eQTL data. Therefore, these results suggest a potential mechanism by which higher methylation levels of cg27583149 may downregulate *VKORC1* expression, which was positively associated with the risk of TN.

*CNOT7* gene expression was associated with a reduced risk of TN in both the discovery and validation sets, and *POMGNT2* gene expression and protein level were associated with an increased risk of TN, which was supported by colocalization (Figure S1C,D).

The corresponding sites of *ATP6V0E2*, cg00753630 and cg24227081, were positively correlated with the risk of TN. *ATP6V0E2* expression was negatively associated with TN risk. The corresponding sites cg00753630 and cg24227081 were negatively correlated with *ATP6V0E2* expression. Therefore, these observations suggest a potential mechanism that higher methylation levels at cg00753630 and cg24227081 may downregulate *ATP6V0E2* gene expression, which was negatively associated with the risk of TN.

To graphically represent the outcomes of our SMR analysis, we generated locus plots depicting the relationships among *ATP6V0E2*, *PLAT*, *VKORC1*, and *CNOT7* methylation, gene expression, and TN (Figures S2–S6). We also provided the effect plots illustrating these associations (Figures S2–S6).

### 3.6. Independent Validation of Prioritized Genes

Multiple validation analyses were conducted to evaluate the five prioritized genes identified from the SMR analysis. In the updated FinnGen R12 dataset, several overlapping signals were observed between the discovery and replication analyses across QTL layers. Notably, expression‐level replication supported three prioritized genes (*CNOT7, PLAT,* and *VKORC1*), with effect directions consistent with the FinnGen R10 discovery results (Table S8).

**FIGURE 5:**
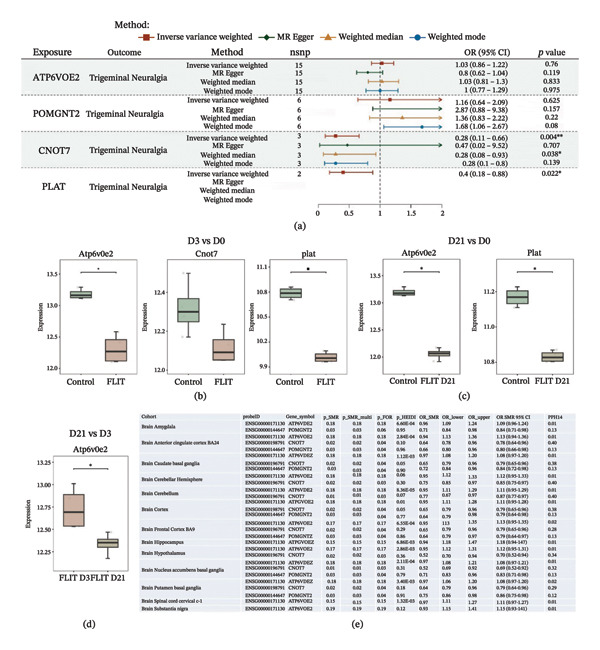
Drug‐target Mendelian randomization and transcriptomic validation of prioritized genes in trigeminal nerve injury models. (A) Drug‐target Mendelian randomization (MR) analysis using blood eQTL variants as genetic proxies for gene expression. Forest plots show the causal estimates for *ATP6V0E2, POMGNT2, CNOT7,* and *PLAT* on trigeminal neuralgia risk across multiple MR methods, including inverse variance weighted (IVW), MR‐Egger, weighted median, and weighted mode models. Odds ratios (ORs) and 95% confidence intervals are presented. (B–D) Validation of gene expression changes in the mouse trigeminal nerve injury dataset (GSE162284). Boxplots show differential expression of prioritized genes at early injury stage (D3 vs D0) (B), late injury stage (D21 vs D0) (C), and between injury stages (D21 vs D3) (D). Expression levels of *Atp6v0e2, Cnot7,* and *Plat* are shown, with significant downregulation observed following injury. FLIT refers to the foramen lacerum–induced trigeminal nerve injury model. (E) Tissue‐specific eQTL associations of prioritized genes across multiple brain regions from the GTEx dataset. ^∗^ *p* < 0.05.

Drug‐target MR analysis was performed using blood eQTL variants as genetic proxies for gene expression. Among the five prioritized genes, *ATP6V0E2, POMGNT2, CNOT7,* and *PLAT* had sufficient instrumental variables for MR analysis. The IVW method identified *CNOT7* (OR = 0.28, 95% CI: 0.11–0.66, *P* = 0.004) and *PLAT* (OR = 0.40, 95% CI: 0.18–0.88, *p* = 0.022) as being negatively associated with reduced TN risk, with consistent effect directions observed in complementary MR models and in agreement with the SMR results (Figure 5A). Heterogeneity, pleiotropy, and outlier analyses indicated no significant violations of MR assumptions (Table S9). Steiger directionality testing supported the inferred direction of effect (Table S10). Additional MR diagnostic plots are provided in Figure S7.

Genes identified from the union of the SMR eQTL and pQTL discovery results were further evaluated in the mouse trigeminal nerve injury dataset GSE162284. In the early injury stage (D3 vs D0), *ATP6V0E2, PLAT,* and *CNOT7* were significantly downregulated with directions consistent with the SMR results. At the later stage (D21 vs D0), *ATP6V0E2* and *PLAT* remained significantly reduced. In the comparison between injury stages (D21 vs D3), *ATP6V0E2* remained significantly altered, suggesting persistent temporal regulation following trigeminal nerve injury (all adjusted *p* < 0.05; Table S11; Figure 5B–D). In the independent dataset GSE256472, *VKORC1* and *ALAD* showed nominal expression changes consistent with the SMR direction, but neither remained significant after multiple‐testing correction (Table S12).

Brain tissue validation using GTEx eQTL data showed that *CNOT7* was significantly associated with TN risk in multiple brain regions, including the cerebellum, basal ganglia, and cortex (P_SMR < 0.05 and P_HEIDI > 0.05). Other prioritized genes did not show consistent associations (Figure 5E).

**FIGURE 6:**
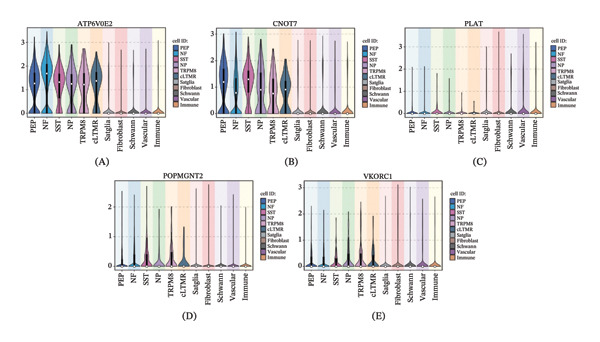
Cell‐type‐specific expression patterns of prioritized genes in trigeminal ganglion single‐cell RNA sequencing data. (A–E) Violin plots showing the distribution of normalized expression levels of *ATP6V0E2* (A), *CNOT7* (B), *PLAT* (C), *POMGNT2* (D), and *VKORC1* (E) across major trigeminal ganglion cell populations in the scRNA‐seq dataset GSE197289. Cell types include peptidergic neurons (PEP), non‐peptidergic neurons (NP), neurofilament neurons (NF), somatostatin neurons (SST), TRPM8 neurons (TRPM8), C‐LTMR neurons, satellite glial cells (Satglia), fibroblasts, Schwann cells, vascular cells, and immune cells.

Finally, scRNA‐seq analysis of trigeminal ganglion tissue (GSE197289) showed heterogeneous cellular expression patterns of the prioritized genes. *ATP6V0E2* and *CNOT7* were mainly enriched in neuronal populations, including PEP, NF, SST, NP, TRPM8, and cLTMR neurons (Figure 6A and B), whereas *PLAT* showed minimal neuronal expression with relatively higher signals in vascular‐associated cells (Figure 6C). *POMGNT2* and *VKORC1* displayed generally low and dispersed expression across cell types (Figure 6D and E; Figures S8 and S9).

### 3.7. Functional Insights From Enrichment and Network Analyses

**FIGURE 7:**
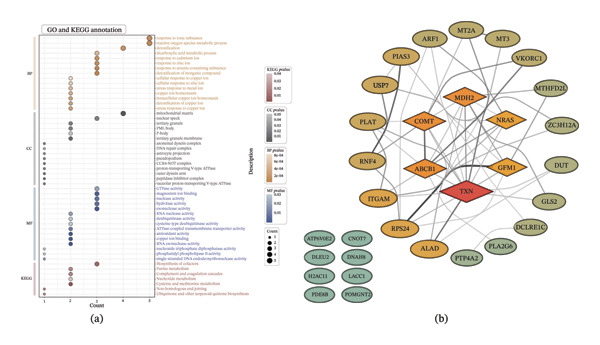
Gene Ontology (GO) and KEGG pathway enrichment analysis. (A) This bubble plot displays significantly enriched terms categorized by GO biological process (BP), cellular component (CC), molecular function (MF), and KEGG pathways. The *x*‐axis (“Count”) and bubble size represent the number of enriched genes in each term. The *y*‐axis (“Description”) lists the specific enriched terms. Bubble color intensity reflects the adjusted *p* value of the enrichment, with distinct color gradients for KEGG (pink/red), BP (orange/brown), CC (gray), and MF (blue/purple) categories. Darker colors indicate lower *p* values, signifying higher statistical significance. (B) Protein interaction network. Key Hub nodes within the interaction network are identified by diamonds, while other candidate target nodes are represented by ovals. Node size and color intensity reflect a quantitative value: increasing from smaller size to larger size, and from greener to orange/red hues, indicating higher values or significance. Line thickness and shade (light to dark) between genes reflect their combined interaction score, with thicker and darker lines indicating higher combined scores.

As mentioned above, 30 genes were identified in the eQTL signals significantly associated with TN risk, while 3 proteins were identified in the pQTL signals using the same criteria. After integrating these results, we obtained 32 unique signals associated with TN risk. GO enrichment analysis indicated that these genes were involved in BP such as response to toxic substances and detoxification. KEGG analysis demonstrated that these genes were involved in pathways such as biosynthesis of cofactors, purine metabolism, and nonhomologous end‐joining (Figure 7A).

Based on the 32 signals associated with TN risk across multiple levels, a PPI network was generated, where the size of each node corresponded to its degree value. Utilizing seven algorithms, we identified the top 6 hub targets (*ABCB1, COMT, GFM1, MDH2, NRAS,* and *TXN*) (Figure 7B, Table S13). These hub genes did not overlap with the SMR‐prioritized genes. The PPI analysis was used to examine interaction relationships among TN‐associated genes.

### 3.8. Drug–Protein Interaction Analysis

To assess the therapeutic potential of the prioritized genes, we performed a druggability analysis using the Enrichr platform (https://maayanlab.cloud/Enrichr/) and its associated Drug Signatures Database (DSigDB). This analysis identified *ATP6V0E2, PLAT, VKORC1*, and *CNOT7*, each of which was mapped to an extensive repertoire of drugs and compounds (Table S14). Among these, *PLAT* and *VKORC1* displayed pronounced druggability, suggesting their potential as candidate therapeutic targets.

## 4. Discussion

This study employed an SMR approach to investigate the association between purine metabolism–related genes and TN. We identified 238 TN‐associated mQTLs (corresponding to 127 genes), 30 TN‐associated eQTLs, and 3 TN‐associated pQTLs, which were further validated by colocalization analysis. Integrated analysis of eQTL and mQTL data suggested that *ATP6V0E2*, *PLAT*, and *VKORC1* may represent prioritized genes associated with TN. Unlike earlier candidate gene studies focusing on ion channel genes such as *SCN9A* [28], this multiomics MR analysis identified metabolic genes, including *ATP6V0E2* and *PLAT*, as potential contributors, likely reflecting the larger GWAS sample size and integration of methylation and transcriptomic data. Although previous studies have reported elevated purine metabolites such as ATP in neuropathic pain [29], this study provides genetic evidence supporting associations between purine‐pathway genes to TN susceptibility.

It is important to note that the prioritized genes identified in this study do not represent core enzymes of purine metabolism but instead appear to influence purine‐related processes through indirect regulatory mechanisms. The initial gene set was derived from GeneCards, which integrates genes with both direct biochemical roles in purine synthesis, degradation, or transport, and genes functionally linked to purine biology through ATP‐dependent processes, oxidative stress, neuroinflammation, and neuronal excitability. Therefore, the prioritized genes may influence purine‐related pathways through indirect regulatory mechanisms rather than direct metabolic activity. To avoid overinterpretation, we distinguish direct biochemical evidence from indirect functional associations when discussing the biological plausibility of each gene.

*ATP6V0E2* encodes a V‐type ATPase subunit, which is essential for cellular acid–base balance, particularly in lysosomal and intracellular environments [30]. *ATP6V0E2* contributes to V‐ATPase‐mediated lysosomal acidification [31]. Impaired lysosomal acidification may disrupt lysosome‐dependent trafficking and exocytosis processes that regulate ion channel localization and ATP release in sensory neurons, thereby influencing neuronal signaling and pain pathways [32]. While there is no direct biochemical evidence that *ATP6V0E2* is involved in purine metabolism, it may influence purine‐related processes indirectly through regulation of ATP synthesis, storage, and release [33], potentially impacting the adenosine signaling pathway. By regulating these metabolic pathways, *ATP6V0E2* may change purine signaling in the central or peripheral trigeminal nerves, leading to abnormal neuronal excitability or inflammatory responses that could aggravate pain conduction. Additionally, our study found that the *ATP6V0E2* gene sites cg00753630 and cg24227081 are negatively associated with gene expression, suggesting that methylation levels at these sites may inhibit *ATP6V0E2*, thereby affecting V‐type ATPase function and changing purine signaling in the trigeminal nervous system, potentially leading to abnormal neuronal excitability or inflammation and intensifying pain conduction. Future research could further explore the mechanisms of *ATP6V0E2* in TN.

*PLAT* encodes tissue‐type plasminogen activator (tPA), an enzyme involved in fibrinolysis that converts plasminogen to plasmin and is closely associated with neurological function and neurotransmission [34]. Despite not being directly involved in purine metabolism, it may influence purine‐related processes indirectly through neuroinflammatory and neuronal regulatory mechanisms. tPA can regulate neuronal excitability through its interaction with NMDAR (N‐methyl‐D‐aspartate receptor) [35], a process that may be linked to ATP release and purine receptor activation, thereby influencing pain sensitivity. Through plasmin generation and extracellular matrix remodeling, tPA is closely related to neuroinflammation [36]. It can induce an inflammatory response in the nervous system by activating microglia and increasing blood–brain barrier permeability [37]. Neuroinflammation is often associated with altered purine metabolism, leading to increased ATP release in an inflammatory state [38], which activates purine receptors, enhances neuronal excitability, and exacerbates TN attacks. Our study found that the methylation levels at *PLAT* gene sites cg17524287, cg24514275, cg12054981, cg17828057, cg01419713, and cg18460120 were negatively associated with *PLAT* gene expression, suggesting that methylation at these sites may inhibit *PLAT*, affecting tPA function and thereby regulating the integrity of the blood–brain barrier, neuronal capabilities, and inflammatory processes. These changes could indirectly influence purine‐related processes and may contribute to TN‐related biological pathway. However, the precise mechanisms require further investigation.

The *VKORC1* gene encodes Vitamin K epoxide reductase complex subunit 1, a key enzyme in the Vitamin K cycle. Although *VKORC1* is not a direct component of purine metabolism, it may indirectly modulate purine‐related pathways through effects on microcirculation, coagulation, and neuroinflammation. *VKORC1* supports the *γ*‐carboxylation of Vitamin K–dependent proteins such as GAS6 [39], which regulates neuroinflammatory responses and microglial activity in the central nervous system [40]. Dysfunction of *VKORC1* can alter the coagulation process, thereby affecting microcirculation and inflammatory responses in the nervous system [41]. When local tissue hypoxia or poor microcirculation occurs, the breakdown of purine nucleotides (such as ATP) accelerates, leading to increased adenosine levels [42]. ATP serves as the primary energy source in cellular energy metabolism, and a deficiency in Vitamin K could impact this process [43], resulting in an imbalance of purine metabolism. Abnormalities in *VKORC1* may regulate pain sensitivity by affecting the generation, release, or degradation of purine compounds (e.g., ATP). Additionally, dysregulation of *VKORC1* can also lead to changes in the coagulation process. Our study found a negative association between the methylation level at the specific *VKORC1* gene site cg27583149 and risk. This suggests that the methylation status at this site may inhibit the *VKORC1* gene, affecting the Vitamin K cycle, and potentially damaging microcirculation, coagulation function, and inflammatory responses in the nervous system, indirectly leading to an imbalance of purine metabolites such as ATP and adenosine. This may finally aggravate the occurrence and perception of pain in TN. However, the precise molecular mechanisms require further investigation.

Enrichment analyses integrate the signals associated with TN risk into a molecular network that aligns well with current pathophysiological models of TN. The gene sets implicated in the cellular response to toxic stimuli, oxidative stress, and nonhomologous end‐joining converge with mounting evidence linking neuropathic pain to neuronal injury, redox imbalance, and neuroinflammation [44, 45]. Notably, the enrichment of purine metabolism among these genes positions purinergic dysregulation as a plausible upstream driver. Specifically, aberrant purine flux may compromise energy‐dependent cytoprotective programs and DNA‐damage checkpoints, thereby amplifying trigeminal nerve injury and central sensitization [46]. Collectively, these findings highlight signals that may be involved in metabolic and neuroinflammatory processes relevant to TN, offering tractable nodes for therapeutic intervention in TN.

PPI analysis identified *ABCB1, COMT, GFM1, MDH2, NRAS,* and *TXN* as potential signals associated with TN pathogenesis. Although these signals did not emerge as causal candidates after multiomics integration, they have been mechanistically associated with nociception. For example, the polymorphisms of *COMT* have been repeatedly associated with susceptibility to diverse chronic pain [47]. In addition, polymorphisms in *ABCB1* are associated with variations in pain severity and the effectiveness of specific medications used for chronic pain, including drug‐resistant TN [48]. Crucially, drug–target mapping places PLAT and VKORC1, both established pharmacologic targets, within a genetically supported regulatory axis for TN. By linking these proteins to TN risk at the genetic level, our study suggests their potential for de novo therapeutic exploitation or drug repurposing. These findings suggest that the prioritized genes may interact with pain‐related factors represented in the PPI network, collectively contributing to TN risk.

Independent validation analyses further supported the relevance of several prioritized genes. Consistent signals across genetic replication, drug‐target MR, and transcriptomic analyses strengthened the relevance of *PLAT* and *CNOT7* in TN‐related BP. Brain eQTL analysis further showed that *CNOT7* was associated with TN risk across several brain regions, suggesting involvement of central regulatory mechanisms. Notably, CNOT7 is a core component of the CCR4–NOT deadenylase complex and regulates mRNA stability and inflammatory gene expression [49], supporting its potential role in TN‐related regulatory processes. In contrast, *ATP6V0E2* showed strong signals in blood‐based analyses and trigeminal nerve injury models but not in brain tissues, indicating possible tissue specificity and suggesting that its effects may be mediated through peripheral metabolic or inflammatory processes. Notably, the GTEx resource does not include trigeminal ganglion tissue. Therefore, the absence of brain‐tissue signals does not exclude potential roles of these genes in the peripheral trigeminal system.

This study employed an SMR approach, integrating data from GWAS, mQTL, eQTL, and pQTL to explore the pathogenesis of TN at multiple levels. This overcame the limitations of single‐omics data analysis and significantly enhanced the robustness and comprehensiveness of the results. Colocalization analysis further supported the associations between GWAS signals and QTLs. However, the study has some limitations. SMR primarily relies on a single top *cis*‐SNP, which differs from conventional multi‐SNP MR approaches. Therefore, the present findings should be interpreted as gene prioritization rather than definitive proof of causality. Although the HEIDI test and colocalization analyses were applied to reduce false‐positive signals driven by linkage disequilibrium, residual horizontal pleiotropy cannot be completely excluded, as genetic variants may influence TN risk through biological pathways independent of the investigated gene expression or methylation signals. The identified genes should therefore be considered high‐confidence candidates that warrant further functional validation. In addition, the limited sample size of the UKBB G6_TRINEU validation cohort, despite successfully validating the findings from the discovery cohort, suggests that the genetic effects on disease risk may be relatively small. Future research using larger datasets is needed to further validate these results. Moreover, the GWAS datasets analyzed were primarily derived from European populations. Replication in other populations will be important to evaluate the generalizability of these results.

Another limitation is that the QTL datasets used in the SMR discovery phase were derived from blood. Because gene regulation can be highly tissue‐specific, blood‐based QTL signals may not fully represent regulatory mechanisms in the trigeminal ganglion or the central nervous system. To partially address this limitation, we integrated brain eQTL validation, trigeminal nerve injury transcriptomic datasets, and trigeminal ganglion scRNA‐seq analyses. However, large‐scale trigeminal ganglion‐specific QTL datasets remain unavailable. Future studies incorporating tissue‐specific QTL resources will be important to further refine these regulatory inferences.

In interpreting the multilayer validation results, it is important to acknowledge the tissue‐ and species‐specific limitations of the datasets used. Blood‐derived QTLs may not fully capture regulatory mechanisms in trigeminal ganglion neurons or other pain‐relevant peripheral tissues, and brain eQTLs from GTEx represent bulk tissue rather than cell‐type‐specific regulatory effects. In addition, the transcriptomic and scRNA‐seq datasets were generated from mouse trigeminal nerve injury models, which differ from human TN in etiology and temporal dynamics. These cross‐tissue and cross‐species differences mean that the validation analyses provide supportive but indirect evidence, and the findings should be interpreted with appropriate caution.

Lastly, the candidate genes identified require further validation through functional experiments to clarify their roles in TN pathogenesis. For example, CRISPR‐mediated knockdown of *ATP6V0E2* in trigeminal neurons followed by electrophysiological assessment could be performed in vitro, whereas *PLAT* inhibition could be tested in TN animal models using behavioral assays such as von Frey filament testing. In addition, *ATP6V0E2* may represent a potential pharmacological target, as V‐ATPase inhibitors such as bafilomycin A1 have been reported [18]. *PLAT*‐related proteins may also have translational potential as diagnostic biomarkers.

The candidate genes and related mechanisms discovered in this study provide new ideas for the diagnosis and treatment of TN. Future research could focus on developing diagnostic kits and targeted therapeutic drugs for TN based on *ATP6V0E2*, *PLAT*, and *VKORC1*. Additionally, a deeper understanding of the genes and proteins involved in TN could guide the development of personalized treatment plans. These findings also serve as potential targets for new drug development, offering new strategies for treating TN and promoting the advancement of precision medicine.

## 5. Conclusions

By using the SMR approach, this study identified multiple purine metabolism–related genes associated with TN, particularly *ATP6V0E2*, *PLAT*, and *VKORC1*, and suggested potential mechanisms related to TN. These findings offer new perspectives for the diagnosis and treatment of TN and may provide a theoretical basis for the development of targeted therapeutic drugs for TN.

## Author Contributions

Jiyan Li, Xiangming Li, Peiyao Li, Yaping Liu, and Libiao Yuan carried out the studies, participated in collecting data, and drafted the manuscript. Simin Shen and Zheng Liu performed the statistical analysis and participated in its design. Xili Zhang, Ying He, and Zhangxiang Huang participated in acquisition, analysis, or interpretation of data and drafted the manuscript.

## Funding

This work was supported by National Natural Science Foundation of China (grant number 82160229), to Zhangxiang Huang; the Yunnan Provincial Education Department Scientific Research Fund Project (2024J0198); the Joint Special Project for Basic Research of Yunnan Provincial Science and Technology Department, and Kunming Medical University (Grant No. 202501AY070001‐014).

## Disclosure

The funders played no role in the design, conduct, or reporting of this study. All authors read and approved the final manuscript.

## Ethics Statement

This article is a Mendelian randomization study. The data for this study were obtained from publicly available databases and published literature data and do not require ethical approval and written informed consent.

## Conflicts of Interest

The authors declare no conflicts of interest.

## Supporting Information

Additional supporting information can be found online in the Supporting Information section.

## Supporting information


**Supporting Information 1** Supporting Information 1. Figure S1. Colocalization results for key gene QTLs and trigeminal neuralgia. A. eQTLs for *PLAT*; B. eQTLs for *VKORC1*; C. eQTLs for CNOT7. D. eQTLs for *POMGNT2*. E. pQTLs for *POMGNT2*. Supporting Information 2. Figure S2. SMRLocusPlot and SMREffectPlot for *ATP6V0E2*. A. SMR Effect Plot for *ATP6V0E2* at cg cg00753630 in mQTL; B. SMR Effect Plot for *ATP6V0E2* at cg24227081 in mQTL; C. The SMR locus plot for *ATP6V0E2* in mQTL; D. SMR Effect Plot of *ATP6V0E2* in eQTL; E. The SMR locus plot for *ATP6V0E2* in eQTL. Supporting Information 3. Figure S3. SMRLocusPlot and SMREffectPlot for *PLAT* in mQTL. A. SMR Effect Plot for *PLAT* at cg17524287; B. SMR Effect Plot for *PLAT* at cg24514275; C. SMR Effect Plot for *PLAT* at cg12054981; D. SMR Effect Plot for *PLAT* at cg17828057; E. SMR Effect Plot for *PLAT* at cg01419713; F. SMR Effect Plot for *PLAT* at cg18460120; G. The SMR locus plot for *PLAT*. Supporting Information 4. Figure S4. SMRLocusPlot and SMREffectPlot for *PLAT* in eQTL. A.SMR Effect Plot of *PLAT*; B. The SMR locus plot for *PLAT.* Supporting Information 5. Figure S5. SMRLocusPlot and SMREffectPlot for *VKORC1*. A. SMR Effect Plot for *VKORC1* at cg27583149 in mQTL; B. The SMR locus plot for *VKORC1* at cg27583149 in mQTL; C. SMR Effect Plot of *VKORC1* in eQTL; D. The SMR locus plot for *VKORC1* in eQTL. Supporting Information 6. Figure S6. SMRLocusPlot and SMREffectPlot for CNOT7 in eQTL. A. SMR Effect Plot of *CNOT7*; B. The SMR locus plot for *CNOT7.* Supporting Information 7. Figure S7. Sensitivity and diagnostic analyses for drug‐target Mendelian randomization. (A) Radial MR plots for *ATP6V0E2* and *POMGNT2* showing SNP‐specific estimates and regression lines for IVW and MR‐Egger models. (B) Scatter plots displaying SNP‐specific associations between genetically predicted gene expression and trigeminal neuralgia risk across MR models (IVW and MR‐Egger) for *ATP6V0E2, POMGNT2, CNOT7,* and *PLAT.* (C) Funnel plots visually assess the symmetry of SNP‐specific causal estimates. (D) Leave‐one‐out analyses showing the influence of each SNP on the overall MR estimate. This analysis was not performed for *PLAT* due to the limited number of instrumental variants. Figure S8. Spatial distribution of prioritized genes across trigeminal ganglion cell populations in single‐cell RNA sequencing data. (A–E) UMAP projections illustrating the expression patterns of *ATP6V0E2* (A), *CNOT7* (B), *PLAT* (C), *POMGNT2* (D), and *VKORC1* (E) in the trigeminal ganglion scRNA‐seq dataset GSE197289. For each gene, the left panel shows normalized gene expression levels mapped onto the UMAP embedding, and the right panel shows annotated cell clusters, including peptidergic neurons (PEP), non‐peptidergic neurons (NP), neurofilament neurons (NF), somatostatin neurons (SST), TRPM8 neurons, C‐LTMR neurons, satellite glial cells, fibroblasts, Schwann cells, vascular cells, and immune cells. Figure S9. Subcluster‐level distribution of prioritized genes in trigeminal ganglion single‐cell RNA sequencing data. (A–E) UMAP projections illustrating the expression patterns of *ATP6V0E2* (A), *CNOT7* (B), *PLAT* (C), *POMGNT2* (D), and *VKORC1* (E) across trigeminal ganglion cellular subtypes in the scRNA‐seq dataset GSE197289. For each gene, the left panel shows normalized gene expression mapped onto the UMAP embedding, and the right panel displays the annotated cell subclusters. These include neuronal and non‐neuronal subtypes such as peptidergic neurons (PEP), non‐peptidergic neurons (NP), neurofilament neurons (NF), somatostatin neurons (SST), TRPM8 neurons, C‐LTMR neurons, satellite glial subtypes, fibroblast subtypes, Schwann cell subtypes, vascular cells, and immune cells.

**Supporting Information 2** Supporting Information Table S1. Finngen_R10_G6_TRINEU cohort mQTLs‐GWAS SMR analysis results. Table S2. mQTLs‐GWAS validation results: mQTLs SMR analysis by UKBB_ G6_TRINEU cohort. Table S3. Finngen_R10_G6_TRINEU cohort eQTLs‐GWAS SMR analysis results. Table S4. eQTLs‐GWAS validation results: eQTLs SMR analysis by UKBB_ G6_TRINEU cohort. Table S5. Finngen_R10_G6_TRINEU cohort pQTLs‐GWAS SMR analysis results. Table S6. pQTLs‐GWAS Validation Results: pQTLs SMR analysis by UKBB_ G6_TRINEU cohort. Table S7. mQTLs‐eQTLs SMR analysis results. Table S8. Overlapping SMR signals between the FinnGen R10 discovery analysis and the FinnGen R12 replication dataset across QTL layers. Rows highlighted in yellow indicate signals corresponding to the five prioritized genes identified in the multiomics integration analysis. Table S9. Drug‐target Mendelian randomization results for prioritized genes associated with trigeminal neuralgia, including heterogeneity, pleiotropy, and MR‐PRESSO sensitivity analyses. Table S10. Instrumental variables for drug‐target Mendelian randomization analysis using blood eQTLs for prioritized genes associated with trigeminal neuralgia, including instrument strength and Steiger directionality test results. Table S11. Differential expression of genes identified from the union of SMR eQTL and pQTL discovery results in the mouse trigeminal nerve injury dataset GSE162284. Genes shown in bold indicate differential expression that is statistically significant (adjusted *p* < 0.05) and directionally consistent with the SMR results. Genes highlighted in yellow correspond to the prioritized genes (*ATP6V0E2, PLAT, VKORC1, CNOT7,* and *POMGNT2*). Differential expression was evaluated across three comparisons: D3 vs D0, D21 vs D0, and D21 vs D3. Table S12. Differential expression of SMR‐identified genes from the union of eQTL and pQTL discovery results in the mouse trigeminal nerve injury dataset GSE256472. Table S13. Hub protein selection based on PPI analysis. Table S14. Full results for the drug–protein interaction analysis for the key prioritized signals.

**Supporting Information 3** STROBE‐MR checklist of recommended items to address in reports of Mendelian randomization studies.

## Data Availability

All data generated or analyzed during this study are included in this article and supporting information files.

## References

[1] Allam A. K. , Sharma H. , Larkin M. B. et al., Trigeminal Neuralgia: Diagnosis and Treatment, Neurologic Clinics. (2023) 41, no. 1, 107–121. 36400550 10.1016/j.ncl.2022.09.001

[2] De Toledo I. P. , Conti Réus J. , Fernandes M. et al., Prevalence of Trigeminal Neuralgia: A Systematic Review, The Journal of the American Dental Association. (2016) 147, no. 7, 570–576.e2. 27017183 10.1016/j.adaj.2016.02.014

[3] Lambru G. , Zakrzewska J. , and Matharu M. , Trigeminal Neuralgia: A Practical Guide, Practical Neurology. (2021) 21, no. 5, 392–402, 10.1136/practneurol-2020-002782. 34108244 PMC8461413

[4] Chen Q. , Yi D. I. , Perez J. N. J. et al., The Molecular Basis and Pathophysiology of Trigeminal Neuralgia, International Journal of Molecular Sciences. (2022) 23, no. 7, 10.3390/ijms23073604. PMC8998776 35408959

[5] Micheli V. , Camici M. , Tozzi G. M. et al., Neurological Disorders of Purine and Pyrimidine Metabolism, Current Topics in Medicinal Chemistry. (2011) 11, no. 8, 923–947, 10.2174/156802611795347645. 21401501

[6] Ma Y. C. , Kang Z. , Shi Y. , Ji W. , Zhou W. , and Nan W. , The Complexity of Neuropathic Pain and Central Sensitization: Exploring Mechanisms and Therapeutic Prospects, Journal of Integrative Neuroscience. (2024) 23, no. 5, 10.31083/j.jin2305089. 38812380

[7] Sautin Y. Y. and Johnson R. J. , Uric Acid: The Oxidant-Antioxidant Paradox, Nucleosides, Nucleotides & Nucleic Acids. (2008) 27, no. 6, 608–619, 10.1080/15257770802138558. PMC2895915 18600514

[8] Chang B. , Wang X. , Chen P. , Ni C. , Niu C. , and Guan H. , Low Serum Uric Acid Levels are Associated With Incidence and Severity in Trigeminal Neuralgia, Neurological Sciences. (2022) 43, no. 10, 6053–6058, 10.1007/s10072-022-06223-4. 35737186

[9] Chang B. , Guan H. , Zhu W. , and Li S. , Low Uric Acid Indicates Risk of Incidence of Trigeminal Neuralgia, Journal of Craniofacial Surgery. (2019) 30, no. 6, e556–e558, 10.1097/scs.0000000000005497. 30939556

[10] Emdin C. A. , Khera A. V. , and Kathiresan S. , Mendelian Randomization, JAMA. (2017) 318, no. 19, 1925–1926, 10.1001/jama.2017.17219. 29164242

[11] Yuan Q. , Sha Y. , Ye R. et al., Machine Learning-Based Identification of kbhb-Affected Tumor Cell Subsets as Prognostic and Therapeutic Targets in Breast Cancer, Journal of Translational Medicine. (2025) 24, no. 1, 10.1186/s12967-025-07555-3. PMC12802299 41382084

[12] Yuan Q. , Zhang H. , Zhang N. et al., BPHL Promotes TNBC Stemness by Resolving R-Loops via POLR2A Lactylation Inhibition and BARD1-Mediated Ubiquitination, Cancer Letters. (2026) 645, 10.1016/j.canlet.2026.218388. 41765360

[13] Wu Y. , Zeng J. , Zhang F. et al., Integrative Analysis of Omics Summary Data Reveals Putative Mechanisms Underlying Complex Traits, Nature Communications. (2018) 9, no. 1, 10.1038/s41467-018-03371-0. PMC5834629 29500431

[14] Skrivankova V. W. , Richmond R. C. , Woolf B. A. R. et al., Strengthening the Reporting of Observational Studies in Epidemiology Using Mendelian Randomisation (STROBE-MR): Explanation and Elaboration, BMJ. (2021) 375, 10.1136/bmj.n2233. PMC8546498 34702754

[15] Võsa U. , Claringbould A. , Westra H. J. et al., Large-Scale Cis- and Trans-eQTL Analyses Identify Thousands of Genetic Loci and Polygenic Scores That Regulate Blood Gene Expression, Nature Genetics. (2021) 53, no. 9, 1300–1310, 10.1038/s41588-021-00913-z. 34475573 PMC8432599

[16] Ferkingstad E. , Sulem P. , Atlason B. A. et al., Large-Scale Integration of the Plasma Proteome With Genetics and Disease, Nature Genetics. (2021) 53, no. 12, 1712–1721, 10.1038/s41588-021-00978-w. 34857953

[17] Consortium G. , The GTEx Consortium Atlas of Genetic Regulatory Effects Across Human Tissues, Science. (2020) 369, no. 6509, 1318–1330. 32913098 10.1126/science.aaz1776 PMC7737656

[18] Salova A. V. , Belyaeva T. N. , Litvinov I. K. , Kharchenko M. V. , and Kornilova E. S. , Deacidification of the Endolysosomal System by the Vesicular Proton Pump V-ATPase Inhibitor Bafilomycin A1 Affects EGF Receptor Endocytosis Differently in Endometrial MSC and HeLa Cells, International Journal of Molecular Sciences. (2025) 26, no. 20, 10.3390/ijms262010226. PMC12563051 41155514

[19] Yang L. , Xu M. , Bhuiyan S. A. et al., Human and Mouse Trigeminal Ganglia Cell Atlas Implicates Multiple Cell Types in Migraine, Neuron. (2022) 110, no. 11, 1806–1821, 10.1016/j.neuron.2022.03.003. 35349784 PMC9338779

[20] Liu Y. , Li B. , Ma Y. , Huang Y. , Ouyang F. , and Liu Q. , Mendelian Randomization Integrating GWAS, eQTL, and mQTL Data Identified Genes Pleiotropically Associated With Atrial Fibrillation, Frontiers in Cardiovascular Medicine. (2021) 8, 10.3389/fcvm.2021.745757. PMC8719596 34977172

[21] Advani J. , Mehta P. A. , Hamel A. R. et al., QTL Mapping of Human Retina DNA Methylation Identifies 87 Gene-Epigenome Interactions in Age-Related Macular Degeneration, Nature Communications. (2024) 15, no. 1, 10.1038/s41467-024-46063-8. PMC10912779 38438351

[22] Morrow J. D. , Glass K. , Cho M. H. et al., Human Lung DNA Methylation Quantitative Trait Loci Colocalize With Chronic Obstructive Pulmonary Disease Genome-Wide Association Loci, American Journal of Respiratory and Critical Care Medicine. (2018) 197, no. 10, 1275–1284, 10.1164/rccm.201707-1434oc. 29313708 PMC5955059

[23] Ghatan S. , van Rooij J. , van Hoek M. et al., Defining Type 2 Diabetes Polygenic Risk Scores Through Colocalization and Network-Based Clustering of Metabolic Trait Genetic Associations, Genome Medicine. (2024) 16, no. 1, 10.1186/s13073-023-01255-7. PMC10777532 38200577

[24] Pairo-Castineira E. , Rawlik K. , Bretherick A. D. et al., GWAS and meta-analysis Identifies 49 Genetic Variants Underlying Critical COVID-19, Nature. (2023) 617, no. 7962, 764–768, 10.1038/s41586-023-06034-3. 37198478 PMC10208981

[25] Zhu Z. , Zhang F. , Hu H. et al., Integration of Summary Data From GWAS and eQTL Studies Predicts Complex Trait Gene Targets, Nature Genetics. (2016) 48, no. 5, 481–487, 10.1038/ng.3538. 27019110

[26] Shi S. , Tian X. , Gong Y. et al., Pivotal Role of JNK Protein in the Therapeutic Efficacy of Parthenolide Against Breast Cancer: Novel and Comprehensive Evidences From Network Pharmacology, Single-Cell RNA Sequencing and Metabolomics, International Journal of Biological Macromolecules. (2024) 279, no. Pt 3, 10.1016/j.ijbiomac.2024.135209. 39244135

[27] Yoo M. , Shin J. , Kim J. et al., DSigDB: Drug Signatures Database for Gene Set Analysis, Bioinformatics. (2015) 31, no. 18, 3069–3071, 10.1093/bioinformatics/btv313. 25990557 PMC4668778

[28] Wang X. , Xia Q. , Li Y. et al., Association of Compression Pressure and SCN8A and SCN9A Gene Polymorphisms With the Recurrence of Balloon Compression-Treated Trigeminal Neuralgia, Neurosurgical Review. (2025) 48, no. 1, 10.1007/s10143-025-03792-8. 41037137

[29] Ermakova E. , Svitko S. , Kabirova A. et al., The Role of Purinergic Mechanisms in the Excitability of Trigeminal Afferents of Rats With Prenatal Hyperhomocysteinemia, Biomolecules. (2025) 15, no. 3, 10.3390/biom15030419. PMC11940108 40149955

[30] Collins M. P. and Forgac M. , Regulation and Function of V-ATPases in Physiology and Disease, Biochimica et Biophysica Acta (BBA)-Biomembranes. (2020) 1862, no. 12, 10.1016/j.bbamem.2020.183341. PMC7508768 32422136

[31] Sun X. , Shu Y. , Yan P. et al., Transcriptome Profiling Analysis Reveals That ATP6V0E2 Is Involved in the Lysosomal Activation by Anlotinib, Cell Death & Disease. (2020) 11, no. 8, 10.1038/s41419-020-02904-0. PMC7445181 32839434

[32] Quick J. D. , Silva C. , Wong J. H. et al., Lysosomal Acidification Dysfunction in Microglia: An Emerging Pathogenic Mechanism of Neuroinflammation and Neurodegeneration, Journal of Neuroinflammation. (2023) 20, no. 1, 10.1186/s12974-023-02866-y. PMC10403868 37543564

[33] Kosmidis E. , Shuttle C. G. , Preobraschenski J. et al., Regulation of the Mammalian-Brain V-ATPase Through Ultraslow Mode-Switching, Nature. (2022) 611, no. 7937, 827–834, 10.1038/s41586-022-05472-9. 36418452 PMC11212661

[34] Varangot A. , Lebatard S. , Bellemain-Sagnard M. , Lebouvier L. , Hommet Y. , and Vivien D. , Modulations of the Neuronal Trafficking of Tissue-Type Plasminogen Activator (tPA) Influences Glutamate Release, Cell Death & Disease. (2023) 14, no. 1, 10.1038/s41419-022-05543-9. PMC9845363 36650132

[35] Lesept F. , Chevilley A. , Jezequel J. et al., Tissue-Type Plasminogen Activator Controls Neuronal Death by Raising Surface Dynamics of Extrasynaptic NMDA Receptors, Cell Death & Disease. (2016) 7, no. 11, 10.1038/cddis.2016.279. PMC5260909 27831563

[36] Tang M.-Y. , Gorin F. A. , and Lein P. J. , Review of Evidence Implicating the Plasminogen Activator System in Blood-Brain Barrier Dysfunction Associated With Alzheimer’s Disease, Ageing and Neurodegenerative Diseases. (2022) 2, 10.20517/and.2022.05. PMC8830591 35156107

[37] Hélie P. , Camacho-Toledano C. , Lesec L. et al., Tissue Plasminogen Activator Worsens Experimental Autoimmune Encephalomyelitis by Complementary Actions on Lymphoid and Myeloid Cell Responses, Journal of Neuroinflammation. (2021) 18, no. 1, 10.1186/s12974-021-02102-5. PMC7897384 33610187

[38] Lin M.-M. , Liu N. , Qin Z. , and Wang Y. , Mitochondrial-Derived Damage-Associated Molecular Patterns Amplify Neuroinflammation in Neurodegenerative Diseases, Acta Pharmacologica Sinica. (2022) 43, no. 10, 2439–2447, 10.1038/s41401-022-00879-6. 35233090 PMC9525705

[39] Aydin N. , Ouliass B. , Ferland G. , and Hafizi S. , Modification of Gas6 Protein in the Brain by a Functional Endogenous Tissue Vitamin K Cycle, Cells. (2024) 13, no. 10, 10.3390/cells13100873. PMC11119062 38786095

[40] Tang Y. , Hu H. , Xie Q. , and Shen J. , GAS6/AXL Signaling Promotes M2 Microglia Efferocytosis to Alleviate Neuroinflammation in Sepsis-Associated Encephalopathy, Cell Death Discovery. (2025) 11, no. 1, 10.1038/s41420-025-02507-8. PMC12144116 40480981

[41] Wallin R. , Wajih N. , and Hutson S. M. , VKORC1: A Warfarin-Sensitive Enzyme in Vitamin K Metabolism and Biosynthesis of Vitamin K-Dependent Blood Coagulation Factors, Vitamins and Hormones. (2008) 78, 227–246. 18374197 10.1016/S0083-6729(07)00011-8

[42] Thauerer B. , Zur Nedden S. , and Baier-Bitterlich G. , Purine Nucleosides: Endogenous Neuroprotectants in Hypoxic Brain, Journal of Neurochemistry. (2012) 121, no. 3, 329–342, 10.1111/j.1471-4159.2012.07692.x. 22335456 PMC3499684

[43] Rajagopal S. , Gupta A. , Parveen R. et al., Vitamin K in Human Health and Metabolism: A Nutri-Genomics Review, Trends in Food Science & Technology. (2022) 119, 412–427, 10.1016/j.tifs.2021.12.012.

[44] Carrasco C. , Naziroǧlu M. , Rodríguez A. B. , and Pariente J. A. , Neuropathic Pain: Delving into the Oxidative Origin and the Possible Implication of Transient Receptor Potential Channels, Frontiers in Physiology. (2018) 9, 10.3389/fphys.2018.00095. PMC5817076 29491840

[45] Testa L. , Dotta S. , Vercelli A. , and Marvaldi L. , Communicating Pain: Emerging Axonal Signaling in Peripheral Neuropathic Pain, Frontiers in Neuroanatomy. (2024) 18, 10.3389/fnana.2024.1398400. PMC11265228 39045347

[46] Tam T. H. and Salter M. W. , Purinergic Signalling in Spinal Pain Processing, Purinergic Signalling. (2021) 17, no. 1, 49–54, 10.1007/s11302-020-09748-5. 33169292 PMC7954904

[47] Mannerak M. A. , Lashkarivand A. , and Eide P. K. , Trigeminal Neuralgia and Genetics: A Systematic Review, Molecular Pain. (2021) 17, 10.1177/17448069211016139. PMC8135221 34000891

[48] Benavides R. , Vsevolozhskaya O. , Cattaneo S. et al., A Functional Polymorphism in the ATP-Binding Cassette B1 Transporter Predicts Pharmacologic Response to Combination of Nortriptyline and Morphine in Neuropathic Pain Patients, Pain. (2020) 161, no. 3, 619–629, 10.1097/j.pain.0000000000001750. 31738228 PMC7039325

[49] Collart M. A. , Audebert L. , and Bushell M. , Roles of the CCR4‐Not Complex in Translation and Dynamics of Co‐Translation Events, Wiley Interdisciplinary Reviews: RNA. (2024) 15, no. 1, 10.1002/wrna.1827. PMC10909573 38009591

